# Fiber orientation distribution from diffusion MRI: Effects of inaccurate response function calibration

**DOI:** 10.1111/jon.12901

**Published:** 2021-06-15

**Authors:** Fenghua Guo, Chantal M. W. Tax, Alberto De Luca, Max A. Viergever, Anneriet Heemskerk, Alexander Leemans

**Affiliations:** ^1^ Image Sciences Institute, University Medical Center Utrecht Utrecht University Utrecht Netherlands; ^2^ Cardiff University Brain Research Imaging Centre, School of Psychology Cardiff University Cardiff UK

**Keywords:** apparent fiber density (AFD), constrained spherical deconvolution (CSD), diffusion MRI, fiber orientation distribution (FOD), fiber tractography

## Abstract

**Background and Purpose:**

Diffusion MRI of the brain enables to quantify white matter fiber orientations noninvasively. Several approaches have been proposed to estimate such characteristics from diffusion MRI data with spherical deconvolution being one of the most widely used methods. Spherical deconvolution requires to define––or derive from the data––a response function, which is used to compute the fiber orientation distribution (FOD). Different characteristics of the response function are expected to affect the FOD computation and the subsequent fiber tracking.

**Methods:**

In this work, we explored the effects of inaccuracies in the shape factors of the response function on the FOD characteristics.

**Results:**

With simulations, we show that the apparent fiber density could be doubled in the presence of underestimated shape factors in the response functions, whereas the overestimation of the shape factor will cause more spurious peaks in the FOD, especially when the signal‐to‐noise ratio is below 15. Moreover, crossing fiber populations with a separation angle smaller than 60° were more sensitive to inaccuracies in the response function than fiber populations with more orthogonal separation angles. Results with in vivo data demonstrate angular deviations in the FODs and spurious peaks as a result of modified shape factors of the response function, while the reconstruction of the main parts of fiber bundles is well preserved.

**Conclusions:**

This work sheds light on how specific aspects of the response function shape can affect the estimated FODs, and highlights the importance of a proper calibration/definition of the response function.

## INTRODUCTION

Diffusion MRI allows to characterize tissue microstructure in vivo and noninvasively by measuring the anisotropic diffusion of water molecules.^1^, ^2^ Diffusion tensor imaging (DTI)^3^ is the most widely used model in clinical studies to relate the diffusion MRI signals to the diffusion characteristics of the underlying tissue. However, DTI is inadequate to estimate the directional information in voxels containing crossing fibers^4^, ^5^ and several methods have been proposed to tackle the crossing fibers problem.^6^, ^7^, ^8^ A commonly used approach to resolve more complex fiber configurations in the brain is spherical deconvolution (SD).^9^, ^10^, ^11^ SD also allows for the extraction of fiber population‐specific microstructural measures derived from the magnitudes of the fiber orientation distribution (FOD) functions, such as apparent fiber density (AFD)^12^ and hindrance‐modulated orientational anisotropy (HMOA).^13^

SD requires an appropriate response function as an input to estimate the FOD.^10^ The response function, representing the diffusion signal for a single‐fiber population (SFP), is ideally calibrated from the acquired diffusion MRI data.^14^, ^15^ With a properly chosen or estimated response function, the corresponding FOD should (1) accurately represent the underlying fiber distribution for individual studies, with minimal occurrences of spurious peaks; and (2) provide consistent intersubject comparison metrics for group studies. In individual subject studies, the voxels containing only SFPs are localized, and an average of the diffusivity characteristics within those voxels is used to represent the subject‐specific response function. An inadequately chosen response function can affect the quantification of FOD characteristics like AFD and HMOA.

In order to compare intersubject AFD, Raffelt and colleagues^12^ chose a response function common to all subjects to minimize the differences between subjects for voxel‐wise AFD comparison. Although a common response function provides consistent scaling in the FOD estimation, which is perhaps suitable in group studies for the comparison of FOD‐derived metrics (e.g., AFD and HOMA),^12^ it is unclear whether this is also optimal to estimate the peak orientation for tractography. Specifically, the use of such a common response function for group‐wise analysis may cause biases in the FOD characteristic estimations for individual subjects. Intuitively, the difference in response function characteristics across healthy subjects is not expected to be large, as response functions are generally averaged from more than hundreds of voxels that are supposed to contain SFPs.^9^, ^10^, ^15^ This was partly demonstrated by Jeurissen and colleagues,^16^ who studied the intersubject response functions of 100 healthy subjects from the Human Connectome Project (HCP)^17^ and observed only subtle differences. Accordingly, it seems justified not to be too concerned about intersubject response function variability in healthy subjects, since either using averaged response functions or individual response functions is not likely to affect the FOD profiles in the HCP dataset. However, although the differences in the response functions of healthy subjects may be small,^16^ this is likely not the case for subjects with some form of pathology. The intersubject signal deviations do raise concern for aging and diseased groups. White matter degeneration as an example may cause the response function not to be optimal for the whole brain, introducing spatially varying discrepancies in the FOD. Besides intersubject differences, there are likely intrasubject interregion differences in the response function estimation.^18^

Previous studies have partially investigated the effect of improperly calibrated response functions on the FOD characteristics and fiber tracking. Tournier^10^ and Dell'Acqua^11^ demonstrated that the choice of the response function could directly affect the FOD peak magnitudes, and thus also derived metrics, such as AFD and HMOA, but would leave their orientations unaffected. Dell'Acqua and colleagues^11^, ^13^ investigated with simulations and in vivo data the effects of various response function changes on the FOD profiles, including variations in the response function, in axonal radius, and in the angle of crossing pathways for the damped Richardson–Lucy (dRL) method. Their paper focused on the effect of the response function on FOD amplitudes and the sensitivity of HMOA to diffusivity changes per fiber population. Parker et al.^19^ studied the FOD peak orientations and the occurrences of spurious peaks in simulations as a function of the response function miscalibration for constrained (C) SD and dRL. The results of that study^19^ demonstrate that sharper response functions resulted in more spurious peaks in the FOD profiles, and that the mismatch of the calibrated‐targeted response functions introduced uncertainty on the main FOD peak orientations. However, the authors used the fractional anisotropy (FA) value as a metric to characterize the response functions, a strategy which is unable to describe the true axial and radial diffusivities in crossing fibers.^20^, ^21^ Changes in FA entangle changes in the axial and radial diffusivities, so that the effects of these two diffusivities could not be studied separately.

In this manuscript, we seek to disentangle the effect of the axial and radial component of the response function on the FOD characteristics and, complementing earlier studies,^19^, ^22^ also aim to quantify their effect on AFD. Changes in pathology are likely reflected in changes in either the axial or the radial diffusivity, which in our study, are represented by the shape and scale factor of the response function. Simulations were designed to explore the effects of the response function shape factor on the FOD properties, including the number of FOD peaks, the FOD peak orientation, the FOD peak magnitude, and the AFD. Additionally, to shed light on the effect on the FOD estimation step of mismatches between the employed response function and the underlying tissue properties, in vivo data from the HCP dataset were used. Pathology was simulated by the changes in the shape and the scale factors to illustrate how the choice of the response function can affect the FOD‐derived metrics and fiber tracking.

## METHODS

In the following sections, we give a brief background on (constrained) SD methods to reconstruct the FOD, outline the simulation experiments, and introduce the shape and scaling factor that characterize the response function, and present the parameter settings used in these simulations. Finally, the in vivo data experiments are described.

### Constrained spherical deconvolution

Recent studies showed that crossing fibers account for over 90% of white matter voxels.^4^ The DTI representation cannot resolve crossing fibers by design and thus provides nonspecific metrics in such voxels. SD approaches^9^, ^10^, ^11^, ^23^, ^24^ overcome this limitation and allow for estimating the FOD for more complex fiber configurations, while retaining reasonable computation and acquisition time compared with other methods.^25^, ^26^, ^27^, ^28^

Constrained spherical deconvolution (CSD) assumes that the diffusion MRI signals can be expressed as the spherical convolution of a fiber response function with the FODs in the spherical harmonics basis, thus also assuming the validity of the response function in all voxels. The response function represents the diffusion‐weighted signal of an SFP. Spherical harmonics form a complete basis on the sphere. The diffusion MRI signal is smooth and can be adequately represented using truncated SH series.^29^, ^30^ In clinical studies, signals with up to 60 gradient directions are generally acquired, limiting the order of the spherical harmonics to 8, which we also adopted in this work.

The FODs are used to infer information on the orientation of the fiber pathways under the assumption that the FOD peak orientations coincide with the underlying fiber directions. In addition to directional information, the magnitudes of the FOD are used to compute additional metrics, such as AFD^12^ and HMOA.^13^ The accurate estimation of FOD peak directions and magnitudes is, therefore, essential for subsequent analysis. In order to suppress the negative values caused by the ringing effect and the sensitivity to noise, the regularization of FOD was proposed^10^, ^24^, ^31^ to improve the conditioning of the deconvolution problem, which is further referred to as constrained SD (i.e., CSD). The regularization step may introduce deviation from linearity into the linear problem of SD, which makes the relation between the response function and the FOD estimation not entirely inversely linear.

### Shape and scaling of response functions

The response function used in the CSD process can be either simulated or derived directly from the data. Following the latter approach, which is more common, voxels that have a high chance of containing SFPs are used to calibrate the response function. A straightforward approach to numerically implement the concept of an SFP is to threshold, for instance FA, above a predefined value. However, the choice of FA threshold is not trivial and can cause inaccuracies in the response function estimation.^15^ A data‐driven method using a recursive calibration framework was proposed to estimate the response function from the subject data in an unbiased way.^15^ This method estimates which voxels contain SFPs by iteratively excluding voxels which do not have a single dominant orientation and updating the estimated response function.

The choice of the fiber response function has potential impacts on the peak directions and magnitudes of the FOD.^13^, ^19^, ^24^ Theoretically, changes in the response function are directly reflected in the FOD estimation, but should affect only peak magnitudes while leaving their orientations untouched.^9^, ^13^ However, in practice, due to the low signal to noise ratio (SNR) level in diffusion‐weighted MRI data, the ill‐posedness of inverse problems and the consequent need for regularization, the effects of the choice of response function on the FODs become less trivial.

Parker et al.^19^ investigated alterations of response function by changing its FA value. Here, we acknowledge that changing the FA affects both the scale and shape of the response function. It is thus not straightforward to disentangle an FOD change into scale and shape effects. The shape component is expected to primarily affect the angular resolution, orientation, and the number of resolved peaks, while the scale component changes the scaling of the FOD, which is important when performing fiber tracking to determine the FOD threshold of the peak detection procedure. To this end, we decompose general changes in the response function into specific changes in shape and scale^11^ and analyze the individual effect of these parameters on the FOD characteristics (i.e., magnitude, AFD, the number of peaks, and peak orientations). The following sections describe how we can achieve such changes in shape and scale of the response functions in the simulated and in vivo diffusion MRI data experiments.

### Simulation experiments

#### Modeling of SFPs and response functions

If the diffusivity *D* associated with the underlying fiber population is expressed by an axially symmetric diffusion tensor, whose first eigenvector is in parallel with the z‐axis in the reference coordinate frame, then *D*(θ,φ) can be written as:^23^

(1) $$D_{\left(\theta ,\varphi \right)}=\left[\begin{array}{ccc} \sin\theta \cos\varphi & \sin\theta \sin\varphi & \cos\theta \end{array}\right]\;\left[\begin{array}{ccc} \beta & 0 & 0\\ 0 & \beta & 0\\ 0 & 0 & \lambda \end{array}\right]\left[\begin{array}{c} \sin\theta \cos\varphi \\ \sin\theta \sin\varphi \\ \cos\theta \end{array}\right],$$

where *α* and *β* are the axial and radial diffusivity of the SFP, (θ,φ) is the polar angle set between the fiber orientation and applied gradient. Given the axial symmetry property of the diffusion tensor, Equation (1) can be simplified as:

(2) $$D_{\left(\theta \right)}=\lambda \;cos^{2}\theta +\beta \;sin^{2}\theta \;=\;\alpha \;cos^{2}\theta +\beta ,$$

where *α* = *λ* – *β* is the absolute difference between the axial and radial diffusivity. For simplicity, if we assume that the signal *S*(θ,φ) from each fiber population is a function of *D*(θ,φ), then the diffusion‐weighted signal *S* can be rewritten as:^3^

(3) $$S_{\left(\theta ,\varphi \right)}=S_{0}\;e^{-bD_{\left(\theta ,\;\varphi \right)}},$$

where *S*_0_ is the nondiffusion‐weighted signal and *b* is the *b*‐value that represents the strength of diffusion weighting. Combining Equations (1)–(3), the diffusion‐weighted signals can be expressed as:^23^

(4) $$S\;\left(\theta \right)=\;\;S_{0}e^{-b\left(\alpha cos^{2}\theta +\beta \right)}=\;S_{0}Ke^{-b\alpha cos^{2}\theta },$$

where *K* = e^–bβ^. Equation (4) highlights the dependency of *S* on the shape factor *α* and the scaling factor *K*, following the definition in previous studies.^11^ In this equation, the scaling factor *K* depends on the radial diffusivity of the fiber response and the applied *b*‐value, representing the isotropic diffusion within the fiber population, whereas the shape factor α depends on the difference between the axial and radial diffusivities, representing the anisotropic diffusion within the fiber population.

#### Modifying the shape and scaling factor of the response functions

Since the response function *R* is intrinsically based on the shape and scaling of the fiber population diffusivities, *R* can be written in the same form as the signal of a fiber population imposed by the gradient at an elevation angle θ with the fiber orientation, which is identical to Equation (4), that is:

(5) $$R\left(\theta \right)\;=S_{0}Ke^{-b\alpha cos^{2}\theta }\;.$$

According to Equation (5), for a given *b*‐value, we can modify (1) the shape factor *α* of the response function, by varying only the axial diffusivity with a fixed radial diffusivity, to keep K constant; and (2) the scaling factor K of the response function, by changing simultaneously the axial and radial diffusivity, to not alter the shape factor *α*. We can then study the effects of *R* on FOD characteristics, by selectively introducing a discrepancy into the shape or the scale of a simulated single‐fiber signal with respect to the response function.

#### Modeling of multifiber populations

We model the diffusion‐weighted signal within a voxel as the sum of multiple compartments measured from each fiber population. Each compartment is assumed to share an identical response function, so the diffusion‐weighted signals are dependent only on the orientations of the fiber populations in the voxel and on data noise. We further assume that there is no exchange of water molecules between fiber populations, and that each SFP can be represented by a signal *S_i_*_(θ)_, where *i* denotes the *i*th fiber population. The signal *S*_DW_ generated by a crossing fiber configuration can then be described by:

(6) $$S_{\mathrm{DW}}=\sum_{i=1}^{n}f_{i}S_{i\left(\theta \right)},$$

where *f_i_* is the signal fraction of each fiber population, *n* is the total number of fiber populations intercrossing the voxel, and *i*(θ) is the angle between the applied gradient and the *i*th fiber population. In our work, we focus on configurations of two crossing fiber populations, but the equations of generating the diffusion‐weighted signals can also be extended to analyze the FOD characteristics for more than two fiber populations.

#### Data analysis

Among the SD frameworks, the CSD approach is implemented in several software packages, including MRtrix,^32^ Dipy,^33^ and ExploreDTI.^34^ In this work, the FOD was estimated with CSD as implemented in ExploreDTI. The FOD peak orientations, which are assumed to reflect the underlying fiber orientations,^9^ and the magnitudes of the FOD peaks, were extracted using a Newton–Raphson gradient descent method.^35^ All FOD peaks that were smaller than an absolute threshold of 0.1 were regarded as contributions from noise and thus discarded to reduce false positives.^36^ All peaks were clustered to the nearest simulated peak directions, by using an angular threshold of 45˚ to determine whether or not two peaks were belonging to the same fiber population. In case of simulating multiple fiber populations, only the estimated FOD peaks closest to the simulated fiber populations were considered. For each simulation, the mean and standard deviation of the following FOD metrics were evaluated:

1. the average difference between the estimated and simulated number of FOD peaks;
2. the angular deviations between the estimated FOD peak orientation and the simulated fiber orientation;
3. the estimated separation angles in case of multiple fiber populations;
4. the FOD peak magnitudes in case of SFPs;
5. the percentage difference of the estimated AFD with respect to the AFD with the reference response function.

The AFD computation was performed as the integral of the FOD magnitudes assigned to each peak, which in the literature is commonly referred to as “lobe.” The calculation of the AFD is similar to what was used in a previous study,^37^ except that we use the gradients generated by the electromagnetic model^38^ to segment the FOD for each fiber population instead of using gradients generated by an icosahedron model.

### Parameter settings

We simulated different fiber configurations with a predefined *b*‐value equal to $3000\;s/mm^{2}$, a set of 60 gradient directions,^38^ and $S_{0}=\;1$. Rician noise (1000 noise instances) was added to the diffusion‐weighted signals to simulate SNR (with respect to S_0_) levels of [50 40 30 20 15 10]. In the first simulation, a single‐fiber configuration was generated with the main diffusion direction along the *z*‐axis, setting $\alpha \;=\;1.2\;\times 10^{-3}\;mm^{2}/s$ and K=0.5 (i.e., *β* ∼ ($0.3\;\times 10^{-3}\;mm^{2}/s$)). In the second simulation, a second fiber population was rotated around the *y*‐axis and combined with the SFP generated in the first simulation to achieve a separation angle ω. Here, we simulated crossing fiber populations with separation angles ω = [90^◦^, 75^◦^, 60^◦^, 55^◦^, 50^◦^, 45^◦^, 40^◦^]. To further explore the impact of inaccurate response functions with respect to the diffusion weighting, a set of multishell diffusion MRI signals was also simulated based on the same HCP dataset gradients, including three diffusion weightings of *b* = 1000 s/mm^2^, *b* = 2000 s/mm^2^ and *b* = 3000 s/mm^2^.

**FIGURE 1:**
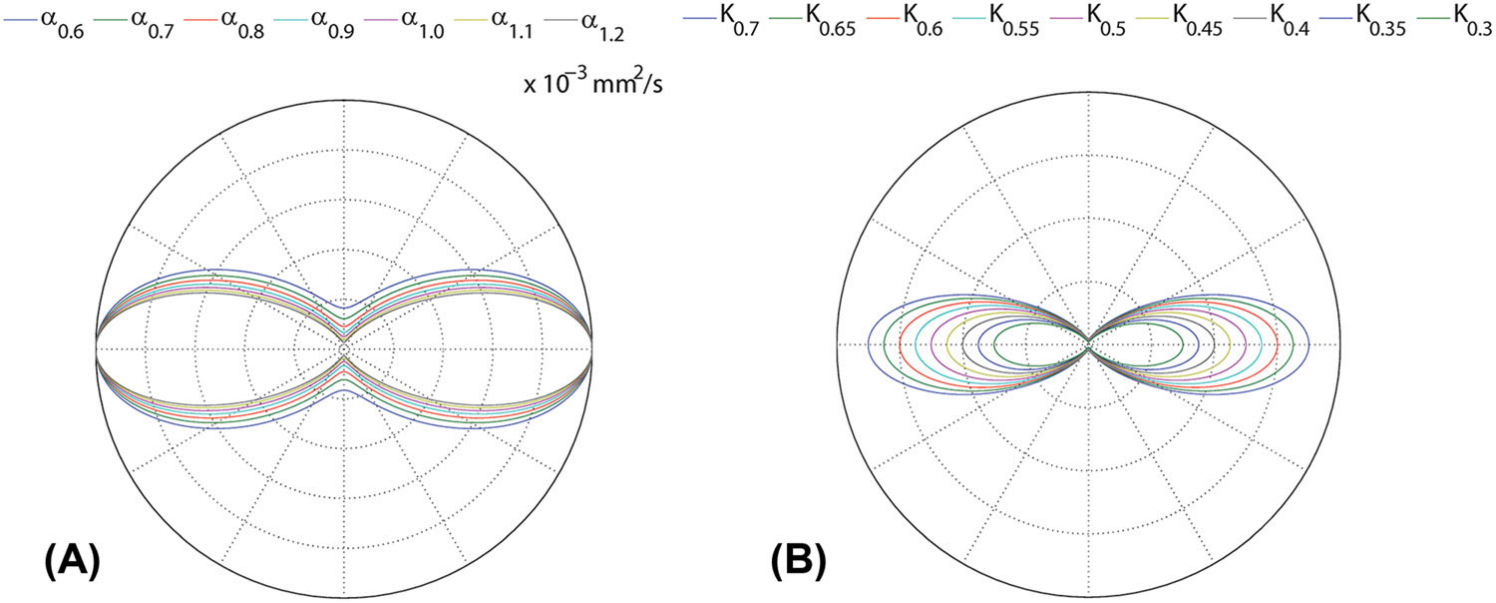
The 2D projection of response functions obtained by changing (A) the shape factor α and (B) the scaling factor K. The shape factors are defined from $0.6\;\times 10^{-3}$ to$\;1.8\;\times 10^{-3}$ mm^2^/s in steps of$\;0.1\;\times 10^{-3}$ mm^2^/s. The scaling factors are varied from 0.7 to 0.3 in steps of 0.05

The 2D projection of two sets of response functions was simulated to achieve (1) different shape but the same scaling factors, by increasing α from $0.6\;\times \;10^{-3}\;mm^{2}/s$ to$\;1.8\;\times 10^{-3}\;mm^{2}/s$ with steps of $0.1\;\times 10^{-3}\;mm^{2}/s$, while keeping K constant (Figure 1A); and (2) the same shape but different scaling factors, by decreasing K from 0.7 to 0.3 with steps of 0.05, while keeping α constant (Figure 1B). In this study, we focus on the shape factor. For multishell simulations, additional data with varying *β* from 0.1 $\times 10^{-3}\;mm^{2}/s$ to 0.5 $\times 10^{-3}\;mm^{2}/s$ (step size 0.05 $\times 10^{-3}\;mm^{2}/s$) while keeping α fixed as 1.2 $\times 10^{-3}\;mm^{2}/s$ were tested.

#### Peak clustering and angular threshold

We clustered the peak directions to make sure that we are always comparing the angular deviations between the simulated fiber orientation and the FOD peak most closely aligned to that orientation. Like in other studies^20^, ^39^, ^40^ that compared axial and radial diffusion characteristics, we also included an angular threshold (e.g., cos (θ) > 0.7, which means approximately θ < 45^◦^) to make sure the correct peaks were being extracted for further evaluations.

#### In vivo data experiments

Diffusion‐weighted MRI data of a single HCP subject were further used to illustrate the effects of ill‐defined response functions on voxel‐wise FOD characteristics and brain tractography. In summary, diffusion‐weighted images were acquired along 90 diffusion gradient directions with a *b*‐value of $3000\;s/mm^{2}$ in addition to 18 nondiffusion‐weighted images, and with an isotropic spatial resolution of 1.25×1.25×1.25 mm^3^. We performed CSD‐based tractography in ExploreDTI with a step size of 1 mm, an FOD threshold of 0.1, an angular threshold of 30^◦^, and seeding points per 2 mm x 2 mm x 2 mm across the whole brain. All the tracts were constructed with deterministic fiber tracking to facilitate data interpretation.

#### Modeling the response function

The reference response function for the in vivo dataset was represented by the diffusion tensor fit to the response function, as estimated with the recursive calibration approach.^15^ The diffusion tensor was used to model the changes in the shape and scaling factor of the response functions. The shape factor α of the response function was modified by +/– $\left[0.1-0.3\;\times 10^{-3}\;mm^{2}/s\right]$, while the scaling factor K was modified by +/– [0.1 − 0.2] to simulate the response function in the case of pathology. The FOD characteristics estimated from the reference response function were set as the baseline values for comparison.

#### Evaluation of in‐vivo data

In analogy with the simulations, we computed the voxel‐wise difference in number of estimated FOD peaks, the angular deviations of the main orientation, and the percentage difference in AFD of the dominant fiber orientation, for all the estimated FODs. The comparisons of number of FOD peaks were computed for the whole brain, whereas the comparisons of angular deviation and AFD were only computed for voxels with FA > 0.2.

Individual white matter fiber bundles were extracted by using the regions of interest as suggested by Wakana.^41^ The segmented fiber pathways include parts of the splenium of corpus callosum (sCC), the genu of corpus callosum (gCC), the cingulum (Cg), the uncinate fasciculus (UF), the corticospinal tract (CST), and the temporal part of the superior longitudinal fasciculus (tSLF). The average FOD characteristics for each fiber bundle were calculated. In addition, FOD characteristics were also derived from CSD using the response functions that were computed from (1) the region with an SFP as identified during the recursive calibration step (referred to as “SFP‐mask”); and (2) the region with voxels for which FA > 0.2 (referred to as “FA‐mask”). White matter templates in ExploreDTI were used in combination with the extracted SFPs to explore the interbundle response functions.

## Results

### Simulations

#### FOD characteristics of SFPs

**FIGURE 2:**
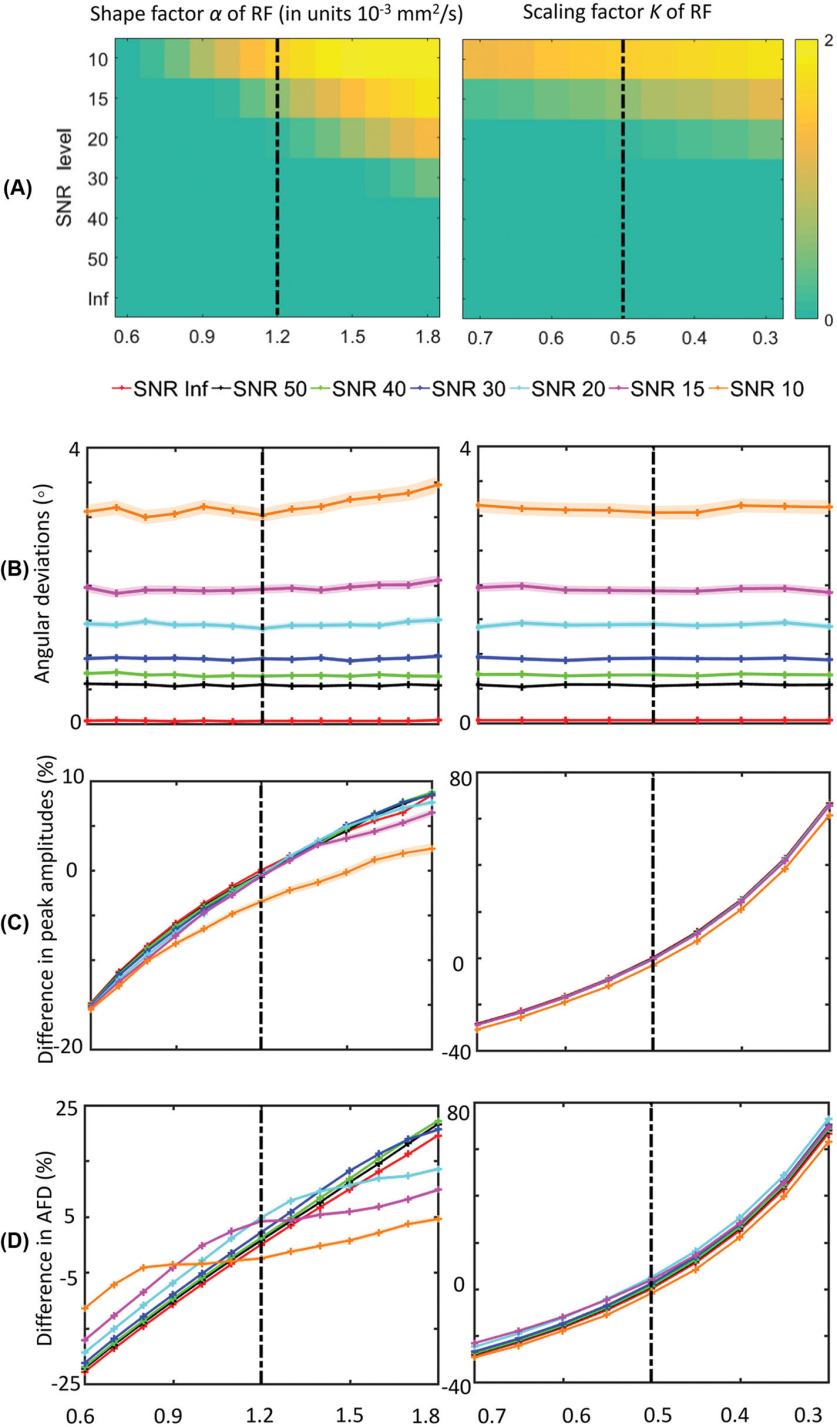
Effect of simulating changes in the response function on fiber orientation distribution (FOD) characteristics for a single‐fiber configuration at different SNR levels. Shape factor *α* and the scaling factor K of the response function are varied at different SNR levels to investigate (A) the introduction of spurious peaks, that is, the average difference between the estimated and predefined number of FOD peaks; (B) the confidence interval (average ± standard error) of the angular deviation of the primary FOD peak; (C) the percentage difference between the amplitudes of the estimated FOD peak and the ground‐truth FOD peak; and (D) the percentage difference between the estimated apparent fiber density (AFD) of the primary fiber population and the ground‐truth AFD. The dashed vertical lines represent the ground‐truth values. Abbreviation: RF, response function

Figure 2 shows the effect of changing the shape factor and the scaling factor of the response function on the FOD characteristics in an SFP. At SNR < 20, the average number of spurious peaks increases when the shape factor increases, but only slightly increases when the scaling factor decreases (Figure 2A). The angular deviation depends mainly on the SNR and to a lesser extent on the shape or scale factor of the response function (Figure 2B). The changes in peak magnitude (Figure 2C) as a function of shape and scaling factor of the response function were not affected by the SNR level. The shape factor has an SNR‐dependent effect, as AFD estimated at low SNR deviate from those estimated at high SNR in the presence of shape factor changes (Figure 2D). Changing the scaling factor from 0.5 to 0.3 or from 0.5 to 0.7 results in around 77% increase or 29% decrease of the peak magnitude, in line with the inverse linear relation between K and FOD. The relation between the scaling factor K and the FOD characteristics aligns with the theoretical expectations, thus for the rest of the study, we only show shape factor‐related results.

#### FOD characteristics of multishell simulations

**FIGURE 3:**
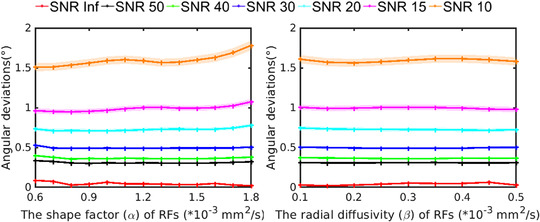
Effect of simulating changes in the response function on fiber orientation distribution (FOD) characteristics for multishell signals. The shape factor *α* and the radial diffusivity *β* of the response function are varied at different SNR levels to investigate the confidence interval (average ± standard error) of the angular deviation of the primary FOD peak. The colors of the lines represent different SNR levels. Abbreviation: RF, response function

Figure 3 shows the effect of changing the shape factor *α* and the radial diffusivity *β* of the response function on the FOD characteristics for multishell simulations. The SNR level clearly affects the estimated FOD characteristics, leading to higher angular uncertainty in correspondence of lower SNR. The angular deviations increase slightly at low SNR when the shape factor of the response functions is bigger than 1.5×10^−3^ mm^2^/s, but does not affected by the radial diffusivity *β*. Overall, the deviations are smaller than those from single‐shell estimation.

#### The effect of the shape factor on angular deviations

**FIGURE 4:**
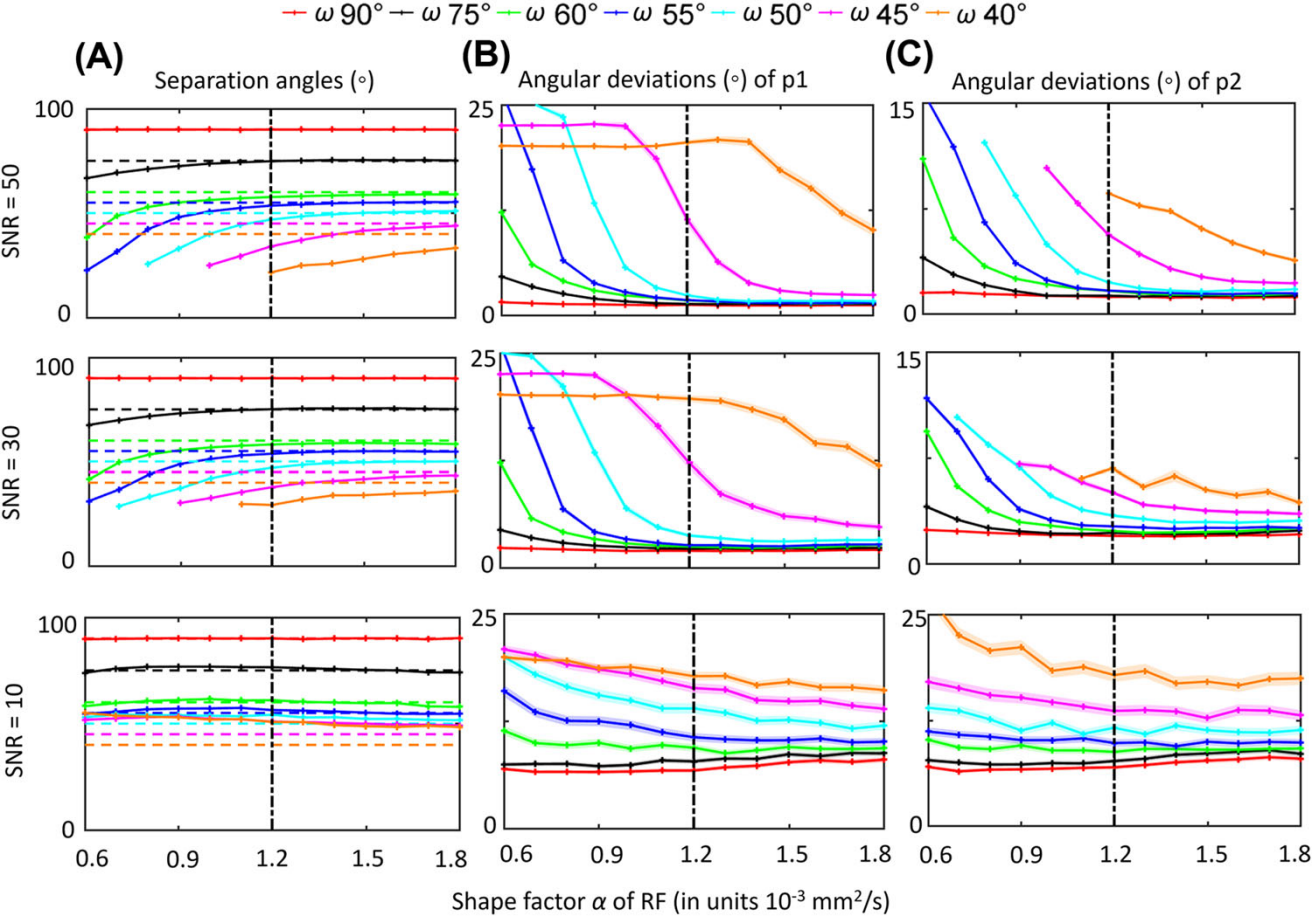
Results of exploring the impact of response functions with different shape factor α on the FOD peaks for crossing fiber configurations simulated with separation angles ranging from 90˚ to 40˚. (A) shows the estimated separation angles between the two primary peaks. Dashed horizontal lines indicate the simulated separation angles. (B) and (C) show the angular deviations between the estimated first (p1) and second (p2) FOD peaks and their corresponding simulated fiber orientations. Solid line interruptions occurred when one of the two peaks was not detected. The means of the estimated values are plotted with the standard error as the shaded areas. The dashed vertical lines represent the ground‐truth response function values. The colors of the lines represent different simulated separation angles. Abbreviation: RF, response function

Figure 4 shows the effect of the shape factor of the response function on the angular characteristics of FOD peaks at SNR = 50, 30, and 10 for crossing fiber configurations with different separation angles. At higher SNR levels (SNR = 30 and 50), lower values of the shape factor generally cause an underestimation of the separation angles, except when the two simulated fiber populations cross orthogonally (i.e., 90^◦^) (Figure 4A). At the lower SNR level (i.e., SNR = 10), the bias in the estimated separation angle due to changes in the shape factor is overruled by the noise itself, especially for lower separation angles. From the observed angular deviations in Figure 4B (the first peak) and Figure 4C (the second peak), in general, the adverse effects of changing the shape factor of the response function on the estimated FOD angular characteristics are more pronounced in smaller separation angles. The underestimation of the shape factor can cause failures in CSD estimation, as shown in Figure 4C, where the second peak is missing at simulated separation angles of 50˚, 45˚, and 40˚.

#### The effect of the shape factor on AFD

**FIGURE 5:**
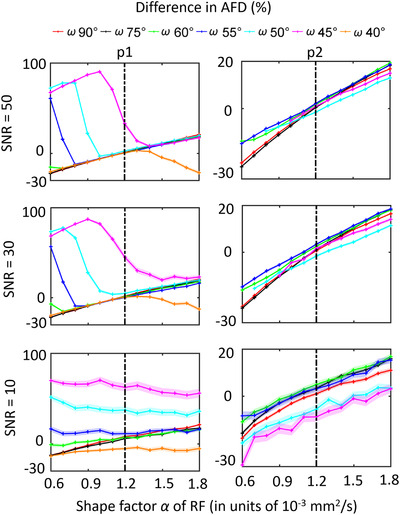
The percentage difference of the estimated apparent fiber density (AFD) of the first peak (p1) and the second peak (p2) in relation to the response function shape factor α at different SNR levels. The quick drop of the AFD difference while increasing the shape factor indicates when constrained spherical deconvolution was able to separate the two fiber populations. The dashed vertical lines represent the ground‐truth response function values. Abbreviation: RF, response function

Figure 5 shows the percentage difference of the AFD of the first and second fiber population in relation to the response function shape factor. In Figure 5A, at SNR 50 and 30, the AFD is high when the shape factor is smaller than 0.8, 1.0, and 1.4×10^−3^ mm^2^/s for the simulated separation angles of 55^˚^, 50^˚^, and 45^˚^, respectively. The AFD values converge to the AFD of the other separation angles as the shape factor increases. As shown in the angular characteristic results (Figure 4), when the response function becomes sharper, the drop points of AFD for small separation angles indicate the boundaries at which CSD is able to separate the two fiber populations. The large difference in AFD for small separation angles (45^˚^−55^˚^) with decreased shape factors can be a confounding factor in intersubject comparisons of AFD studies, which will be discussed further. At SNR 10, the AFD differences are more related to noise than to the shape of the response function for smaller separation angles (below 60^˚^). As for the second peak (Figure 5B), the AFD can change from −30% to 20% when the shape factor was modified from −50% to 50%, respectively.

### In vivo HCP dataset

#### FOD characteristics of white matter

**FIGURE 6:**
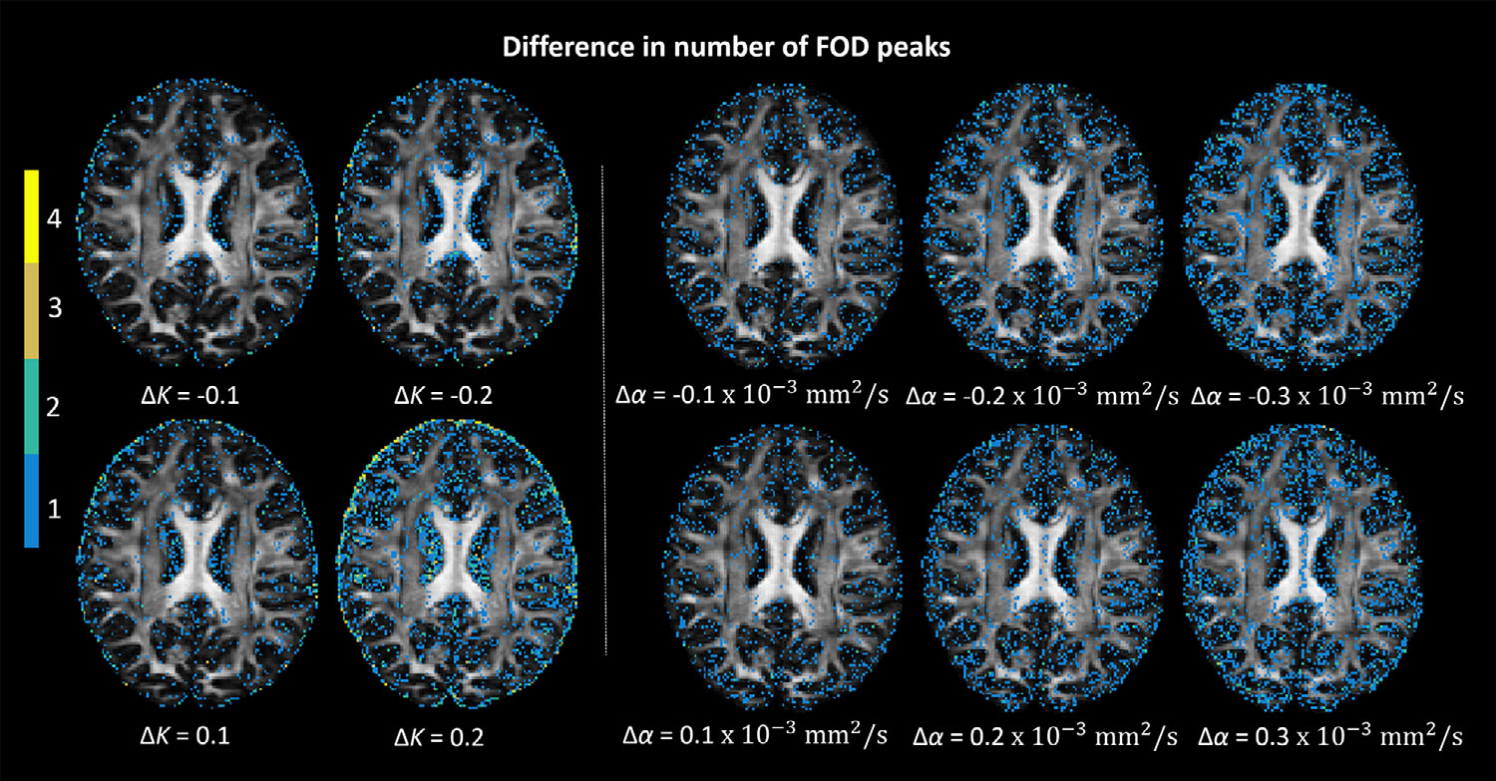
The difference between the number of fiber orientation distribution (FOD) peaks estimated with the tensor‐based response function and the number of FOD peaks computed with the response function modified according to certain changes in scaling (ΔK) and shape (Δα) factors. The background is an axial view of the fractional anisotropy map. The peak number difference mostly occurs in gray matter and cerebrospinal fluid areas, and crossing fiber regions for white matter, as indicated by the colormap. In regions with single‐fiber populations (e.g., middle parts of the corpus callosum), spurious peaks are hardly present

In this section, we present the effect of changing the shape factors of the response function on FOD characteristics for an axial slice of the HCP dataset. The difference in number of FOD peaks per voxel is shown in Figure 6. Differences are typically seen in areas with partial volume effects and with mostly a peak number difference value of one. When the difference in shape factor, denoted by Δα, increases by 0.1 $\times 10^{-3}\;$ to 0.3 $\times 10^{-3}\;$mm^2^/s, one can see the increase in occurrence of peak number deviations, such as, for instance, in the mid‐sagittal regions of the corpus callosum. With the increase of difference in scaling factor, denoted by ΔK, regions containing cerebrospinal fluid (CSF) showed higher peak number differences than regions with white and gray matter.

**FIGURE 7:**
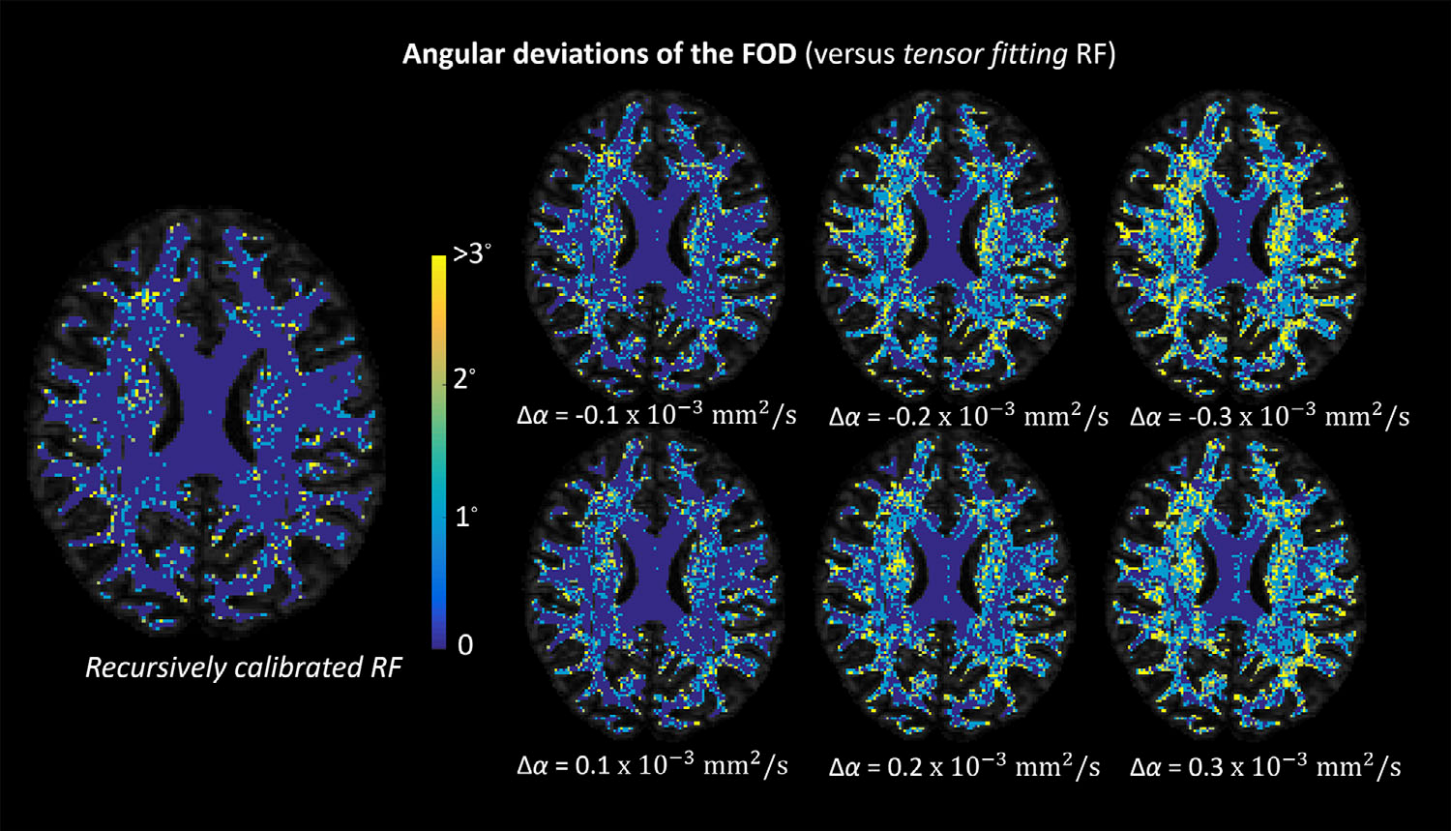
The angular deviations between the FOD peaks estimated with the recursive calibrated response function, the tensor‐fit of the response function, and the FOD peaks estimated with the response function modified according to certain changes in shape (Δα) factors. The background is an axial view of the fractional anisotropy (FA) map and, for clarity, the angular deviations are shown only in regions where FA > 0.2. Most angular differences are in the range of 0–3^˚^. Similar to the results of spurious peaks shown in Figure 6, angular deviations are larger in regions with crossing fiber populations than regions with single‐fiber populations, such as the middle part of the corpus callosum. Abbreviation: RF, response function

The left panel of Figure 7 shows the angular differences between the primary FOD peak computed with the recursive calibrated response function and the FOD peak obtained with the tensor‐fit to the recursive calibrated response function. Although the angular deviation is close to zero in most areas, there are some voxels in the crossing fiber regions that show 1˚–3˚ angular deviations of the main FOD peak. Figure 7 right shows the angular difference between the primary FOD peak, computed with the tensor‐fit to the recursive calibrated response function, and the FOD peak obtained with the modified shape factors of the response function. In general, regions containing crossing fibers are affected most when modifying the shape of response functions, with angular deviations of the main FOD peak of more than 3^˚^. Increasing the magnitude of Δα resulted in larger angular deviations.

**FIGURE 8:**
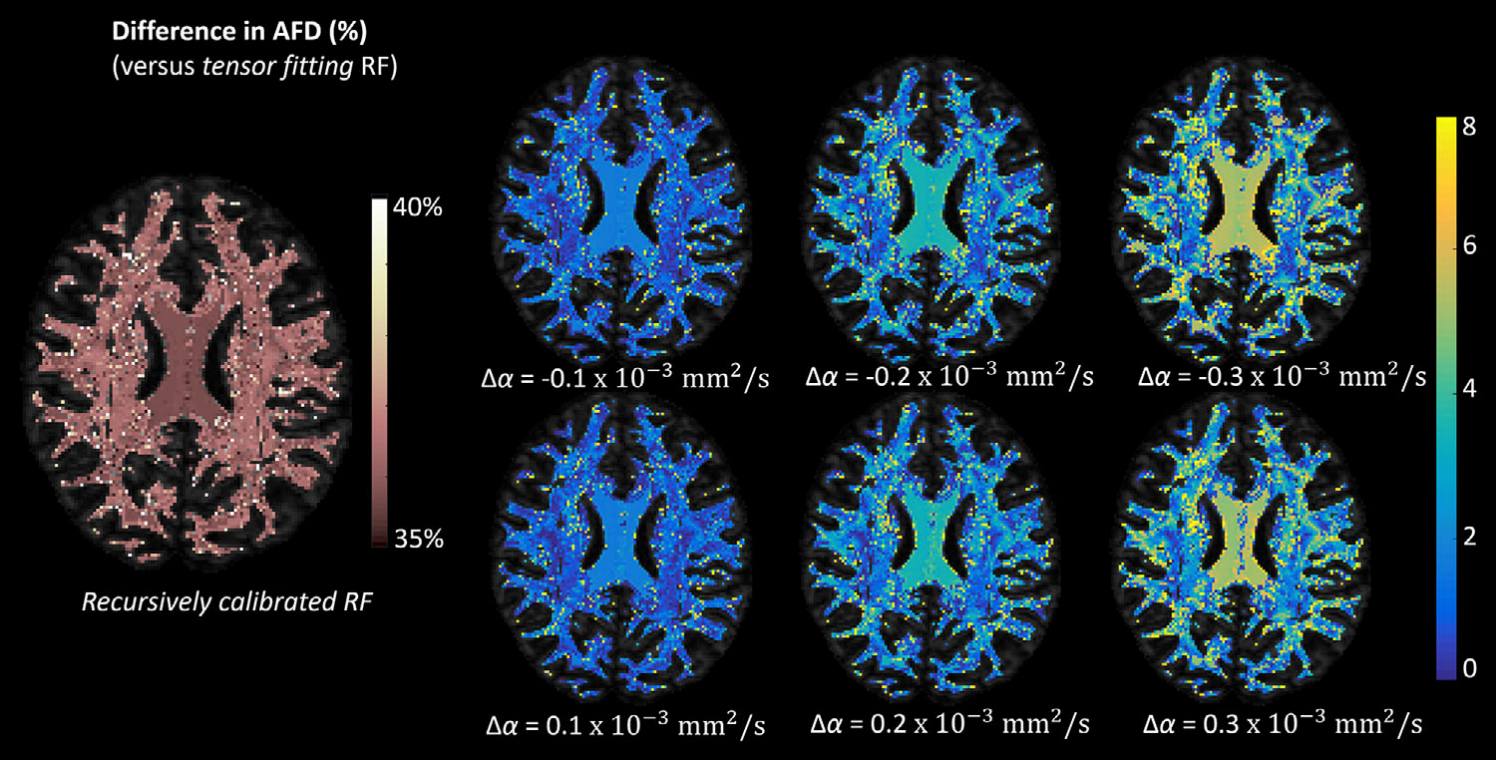
The percentage difference of the apparent fiber density (AFD) between the FOD peaks estimated with the recursive calibrated response function, the tensor‐fit of the response function, and the FOD peaks estimated with the response function modified according to certain changes in the shape (Δα) factors. The background is an axial view of the fractional anisotropy (FA) map and, for clarity, the AFD percentage differences are shown only in regions where FA > 0.2. AFD differences are mainly located in the corpus collosum area. Abbreviation: RF, response function

The left column of Figure 8 shows the voxel‐wise AFD difference for the dominant fiber direction between the FOD estimated with the recursive calibrated response function and the FOD peak obtained with the tensor‐fit to the recursive calibrated response function for the HCP dataset. The percentage difference of AFD is around 36% in most brain areas, with some extent of heterogeneity within 5% across the brain that relates to the variations in the shape factor *α* of the response function. Figure 8 right shows the voxel‐wise AFD difference for the dominant fiber direction between the FOD estimated using the tensor‐fit to the recursive calibrated response function and the FOD obtained with the modified shape factors of the response function for the HCP dataset. When changing the shape factor with −0.3 $\times 10^{-3}\;$ to 0.3 $\times 10^{-3}\;$mm^2^/s, the highest AFD differences (around 6–8%) were observed in areas with an SFP, such as the corpus callosum. Larger changes of the magnitude of the shape factor *α* make the AFD difference more heterogeneous across the brain.

#### Effect on fiber tractography

**FIGURE 9:**
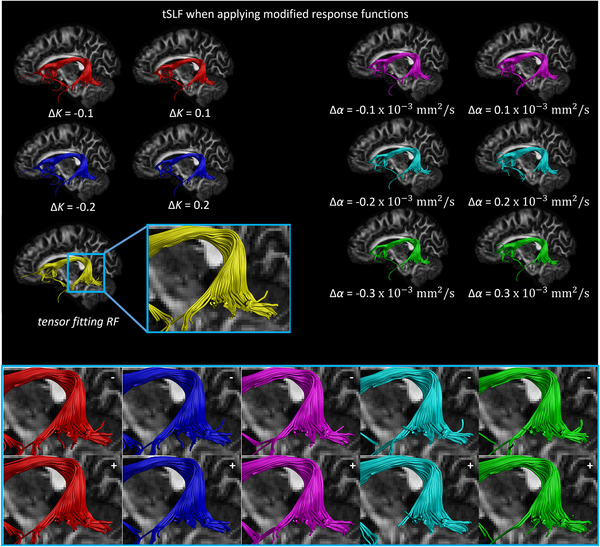
The temporal part of the superior longitudinal fasciculus (tSLF) reconstructed with the FODs estimated using the tensor‐fit to the recursively calibrated response function (yellow), and the tSLF from the same regions of interest reconstructed with FODs estimated using the modified response functions. The other fiber bundles (shown in red, blue, cyan, magenta, and green) indicate the effect of changing the scaling (ΔK) and shape (Δα) factors of the response function on the trajectory of the tSLF. Subtle differences in how the fiber trajectories terminate in the temporal lobe are shown in the enlarged area (zoomed areas; the “+” and “–” indicate increase and decrease in the scaling and shape factors, respectively), while main parts of the tracts are reserved. Abbreviation: RF, response function

**FIGURE 10:**
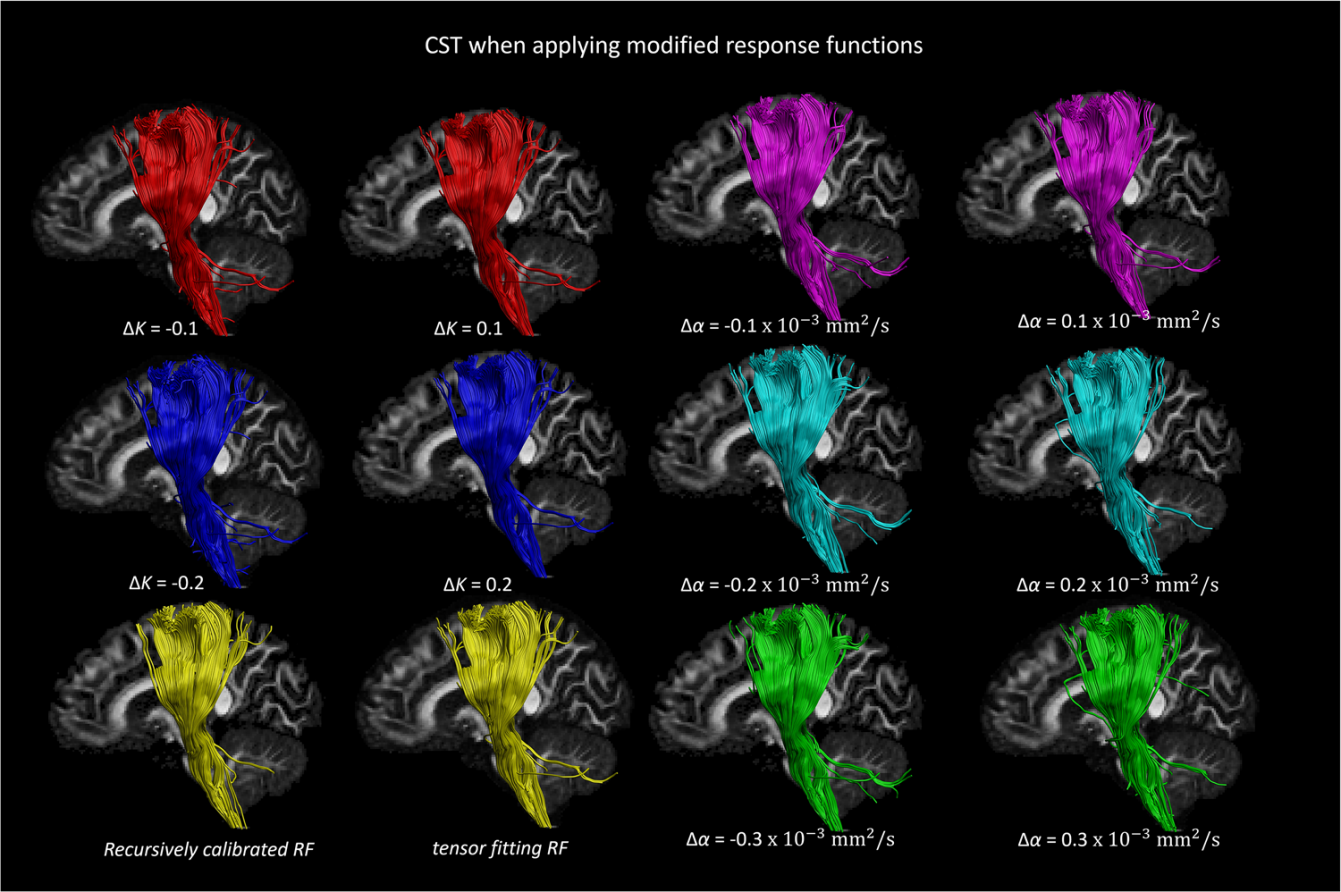
The corticospinal tract (CST) reconstructed with the FODs estimated using the tensor‐fit to the recursively calibrated response function (yellow), and the CST from the same regions of interest reconstructed with FODs estimated using the modified response functions. The other fiber bundles (shown in red, blue, cyan, magenta, and green) indicate the effect of changing the scaling (ΔK) and shape (Δα) factors of the response function on the trajectory of the CST. The “+” and “–” indicate increase and decrease in the scaling and shape factors, respectively. Abbreviation: RF, response function

Figure 9 shows the effect of changing the scale and shape factors of the response function on the reconstruction of the pathways of the tSLF. The reference trajectories (shown in yellow) are computed with the recursive calibration method. Only small differences can be observed for the main part of the reconstructed tracts. Changing the response function (Figure 9) causes subtle changes in the majority of the tracts, with the ends of the trajectories where the tSLF enters the frontal and temporal lobes varied slightly (see enlarged regions in Figure 9). Figure 10 shows the effect of changing the scale and shape factors of the response function on the reconstruction of the pathways of the CST.

**FIGURE 11:**
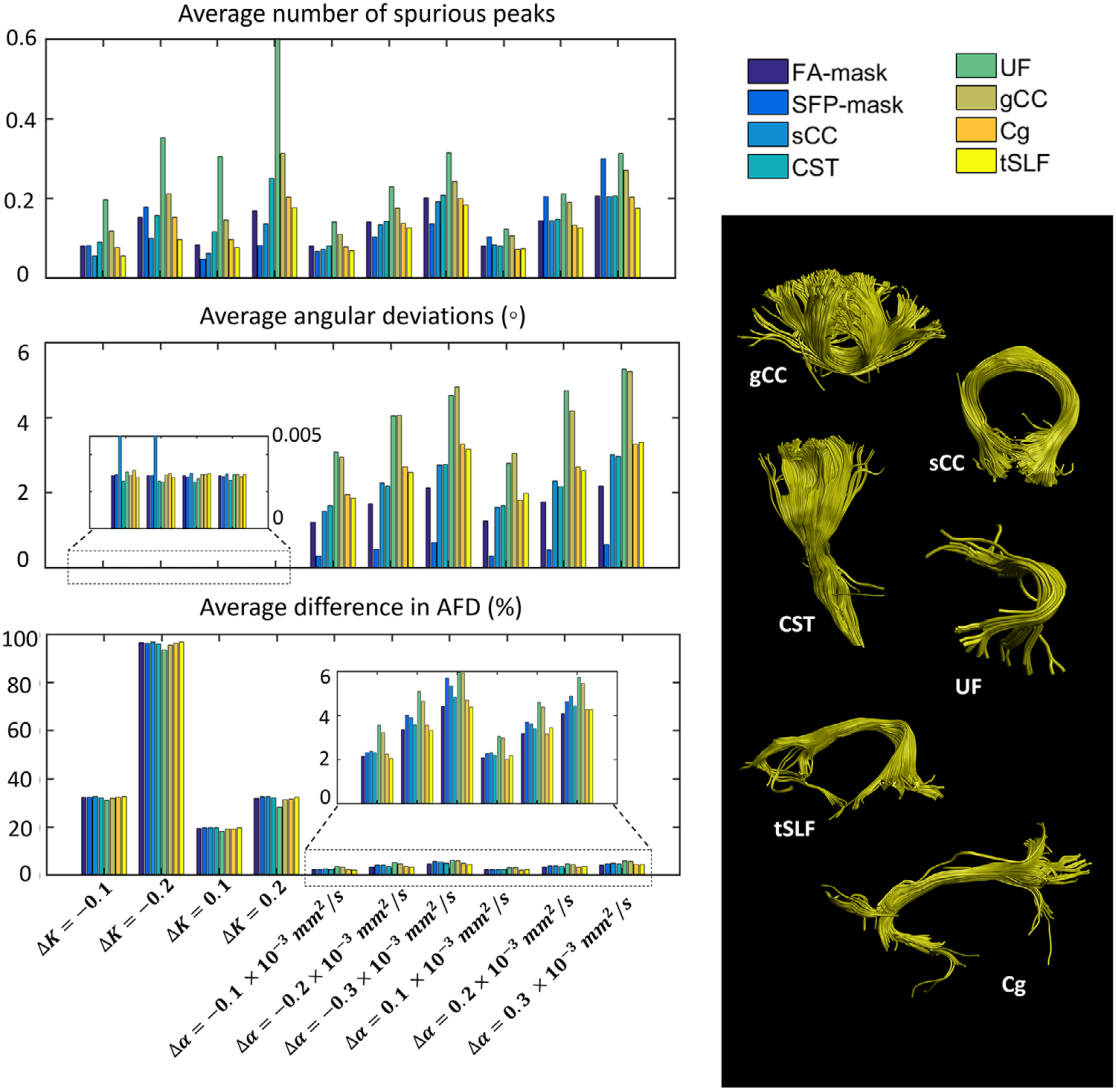
The average number of spurious peaks, the average angular deviations, and the average percentage differences in AFD of the first fiber population for the fractional anisotropy (FA)‐mask, single‐fiber population (SFP)‐mask, and the selected fiber bundles (shown on the right) when a modified response function was used in comparison to the original tensor‐fit to the recursive calibrated response function. The effect of the changes in the scaling (ΔK) and shape (Δα) factors of the response function on the selected fiber bundles is reflected in the different color encoding. Abbreviations: Cg, cingulum; CST, corticospinal tract; gCC, genu of corpus callosum; sCC, splenium of corpus callosum; tSLF, temporal part of superior longitudinal fasciculus; UF, uncinate fasciculus

Figure 11 shows the FOD characteristics for the FA‐mask, SFP‐mask, and the extracted fiber bundles (gCC, sCC, CST, UF, Cg, and tSLF). From all three FOD characteristics (i.e., spurious peaks, angular deviations, and AFD percentage differences), we can spot a similar trend for all the bundles and masks with respect to the changes in the shape and scaling factors of the response function. Overall, the UF has the highest average number of spurious peaks. The lowest average angular deviations of the first FOD peak can be seen for the SFP‐mask. Furthermore, the alterations of the shape factor of the response function can cause angular deviations up to 6˚, while the alterations of the scaling factor hardly cause any angular differences in the masks or the selected fiber bundles, as expected (see the enlarged plot). Finally, the differences in AFD are relatively homogenous across the extracted fiber bundles and masks as a function of changing the shape or scaling factors.

Table 1 shows the shape (*α*) factors, scale (*K*) factors, and the FA of the response functions estimated from single‐fiber voxels of several fiber bundles and SFPs of an HCP subject, along with the percentage differences (Δ*α*, Δ*K*, and ΔFA) compared to those calculated from the recursive calibrated response functions from the single fibers across the brain. The forceps show higher shape, scale factors, and FA, while the inferior fronto‐occipital fasciculus, inferior longitudinal fasciculus, and the superior longitudinal fasciculus show lower shape, scale factors, and FA as compared to SFPs. Other fiber bundles also show differences in the shape and scale factors when comparing to the values of SFPs.

**TABLE 1:** Response functions across fiber bundles

Shape/scale factors of fiber bundles								
	SFP	Cingulum	CST	Forceps	IFOF+ILF	SLF	ATR	Uncinate
K/ΔK	0.27	0.277/+2.44%	0.266/−1.41%	0.314/+16.2%	0.24/−11.45%	0.23/−14.87%	0.265/−2%	0.262/−3.04%
*α* (x10 ^−3^ mm^2^/s)/Δ*α*	1.043	1.054/+1.08%	0.996/−4.51%	1.136/+8.94%	0.948/−9.12%	0.91/−12.71%	1.057/+1.32%	0.983/−5.7%
FA/ΔFA	0.65	0.66/+1.06%	0.64/−2.17%	0.71/+7.73%	0.61/−7.24%	0.59/−9.95%	0.65/−0.16%	0.63/−3.12%

*Note*: The shape (*α*) factors, scale (*K*) factors, and fractional anisotropy (FA) of the response functions estimated from different brain structures and the percentage differences (Δ*α*, Δ*K*, and ΔFA).

Abbreviations: ATR, anterior thalamic radiation; CST, corticospinal tract; IFOF ILF, inferior fronto‐occipital fasciculus and inferior longitudinal fasciculus; SFP, single‐fiber populations; SLF, superior longitudinal fasciculus; Uncinate, uncinate fasciculus.

## DISCUSSION

In this work, we investigated the effect of changing response function properties on the FOD characteristics using numerical simulations and data from the human connectome project. We have shown how miscalibration of the response function, as defined by adjusting the shape factors, can introduce a bias in the orientation and magnitude of fiber population peaks. Our findings demonstrate that CSD is prone to produce spurious FOD peaks in the presence of miscalibrated response functions, especially in combination with data with insufficient SNR levels. The occurrence of such spurious peaks, however, mainly happens in CSF and cortical areas, thus the fiber pathway reconstruction was only subtly affected. Overall, in agreement with former studies, spurious peaks are introduced due to overestimating the shape factor of the response function, while underestimating the shape factor will result in lower angular resolution of the FOD lobes.^13^, ^19^ Proper tuning of the response function is, therefore, beneficial to achieve an optimal balance between increasing the angular resolution and minimizing the number of spurious peaks, especially for smaller separation angles (i.e., below 60^˚^) and at low SNR levels. Further, AFD estimation can be influenced by the choice of response function.

In Figure 2, we see the FOD magnitudes and RF scaling factors are inversely related to each other. As this was indicated in the formula in CSD estimation,^10^ from the simulations we see that this relation was maintained in the presence of noise and regularization. In Figure 11 of in vivo data study, varying the scaling factors did not affect the angular deviations along the fiber bundles. At SNR levels of 30 and 50, the FOD characteristics are consistently affected by the choice of the response functions, while at SNR of 10, noise is the dominating factor that affects the FOD properties. In particular, using a sharper response function for separation angles below 50˚ can potentially increase the angular resolution of CSD and can, therefore, improve the estimation of the number of peaks in crossing fibers. The shape of the response function was reported to vary with axonal injury and brain maturation, whereas the scaling factor was observed to change as a result of demyelination, axonal diameters, and axonal density changes.^13^, ^42^ This implies that in brain regions affected by disease, applying CSD with a response function determined by healthy white matter data can result in unreliable estimates of FOD characteristics.

### The interpretations of the response function

The response function represents the expected signal profile in voxels containing a single white matter fiber orientation, which is supposed to be comparable among healthy subjects. In CSD implementations, such as MRtrix and ExploreDTI, the response function is estimated per subject to capture individual‐specific properties of the white matter structure in single subject studies, in particular for the purpose of fiber tractography. In group studies on voxel‐wise diffusion metrics, however, how to define or choose the response function requires further thought. Specifically, when measuring the response function from pathology regions or brains, whether a response function should be estimated from healthy tissues or pathological tissues need to be considered. In pathological structures, the response function may have lower scaling and shape factors due to, for example, loss of axons. When a healthy tissue response is used, the AFD will be proportionally smaller and reflects the lower axon density. In this case, the response function measured from healthy tissues can be used as the measuring unit for the deconvolution approaches to identify FOD characteristics.

The intersubject response function deviations can have several reasons, including but not limited to axon density, axial diameter, membrane permeability, and myelination.^43^ Considering the achievable time and resolution of diffusion MRI, the response function relates closely to the intra‐axonal volume and the intrinsic axonal signal. Intrinsic signal changes can be caused by, for example, changes in axon diameter distribution, or additional constituents with non‐negligible diffusion signals, such as pathology. However, when performing comparisons of FOD metrics, differences observed in AFD will normally be interpreted as differences in intra‐axonal signal fraction. On a macroscopic level, changes in intra‐axonal signal fraction will be indistinguishable from changes in partial volume of white matter. Therefore, regional pathology and partial volume are the main factors related to response function changes when exploring the FOD characteristics.

In case of regional pathology, the response function should be derived from global estimation or healthy tissues, instead of from the region itself. The response function then does not vary spatially to reflect local tissue properties, but serves as the measure unit for comparing, for example, AFD. The degraded AFD in presence of a global response function reflects the axon degeneration caused by pathology. As we mentioned in the introduction, this raised concern on whether it is optimal as well for tractography and whether it affects the accuracy of local FOD estimation. Since pathology may cause a reduction in the shape factor in the measured diffusion signal, it could be argued that the healthy tissue response function would have a shape factor that is too high for the estimation of the FOD in this region. A response function with a larger shape factor could result in more spurious peaks on the one hand, but also preserve or increase angular resolution on the other hand. This means the tractography performance would be preserved, as also shown in the in vivo tractography illustrations in Figures 9 and 10.

For clinical applications which aim at detecting regional pathology within every single subject, a global response function can be used in the FOD estimation. As such, the same measuring unit, that is, the response function is used, and the regional tissue characteristic differences of the shape and scaling factors would be reflected in the corresponding deviations in the FOD metrics.

### Effect of the separation angle between crossing fiber populations

The extent to which the FODs will be affected by the response function depends largely on the separation angle between crossing fiber populations (Figure 4). More orthogonally crossing fiber orientations are less sensitive to response function changes, as originally suggested in the SD paper.^9^ In voxels containing crossing fiber configurations with smaller separation angles (e.g., below 60^˚^), the average angular deviations and their variance increase rapidly with lower shape factors of the response function. Higher shape factors of the response function result in smaller bias in the computation of the FOD peak orientations than the underestimation of the shape factor (Figures 4 and 5).

### Adverse effect of the shape factor on CSD angular resolution

For fiber populations with separation angles below 55^˚^, CSD fails to estimate the correct number of peaks when response functions with a lower shape factor are employed, leading to artificially higher AFD values (Figure 5). As FOD peaks merge together when the shape factor is further decreased, the AFD becomes close to the integral of the total FOD amplitudes within the voxel. This is shown in Figure 5 for simulated separation angles between 45˚ and 55˚. For these relatively small separation angles, the large AFD difference is caused by the limited angular resolution of CSD with the simulated settings. Previous studies^44^ report AFD as a more sensitive diffusion marker in traumatic brain injury than the traditional metrics. However, one should be aware that these changes in AFD in the presence of pathology could result from the limits of CSD angular resolution that was affected by the shape of the response functions. The bias resulting from using a group averaged response function instead of a per‐subject response function can cause differences in the number of fiber populations, thus local diffusivities alteration within voxels.

### Effect of FOD angular deviations on fiber tracking

Because of the stepwise nature of fiber tractography, the reconstructed fiber pathways accumulate angular errors during their propagation. If a systematic angular deviation is introduced in the FOD estimation step, the whole fiber bundle will accumulate geometrical distortion. Conversely, if the angular deviations are not stepwise systematic along the reconstructed fiber pathway, the FOD estimation deviations will affect voxel‐wise characteristics, such as AFD and the number of fibers. In our results, we did not observe relevant errors in the main part of the fiber bundles, but subtle differences at the edges (Figure 9). With the in vivo HCP data, only minor changes in the tSLF trajectories are detected when using the modified response functions with different shape factors, suggesting good robustness of the streamlines reconstruction to response fiber miscalibrations. The termination of fiber pathways passing through crossing regions can be affected;^15^ however, for the two fiber bundles of tSLF and CST estimated from HCP dataset, the influence is more pronounced on the ending parts of the trajectories.

### Interbundle response functions

Previous studies^45^ reported interbundle differences in the response function along with other DTI metrics, with a conclusion that interbundle fiber response shows larger differences than intersubject fiber response. The results were partially validated in a recent histology study,^46^ that when response functions derived from different regions are applied, the reconstructed FODs have similar shapes and orientations but the signal fractions of fiber populations are different, that is, a potential difference in AFD. In Table 1, we show the shape and scale factors of the response functions that were estimated from several fiber bundles in the brain. In comparison to using the response function that recursively calibrated from SFPs, different brain structures show some deviations in the shape and scale factors, with around 11% decrease in the inferior fronto‐occipital fasciculus and inferior longitudinal fasciculus, 14% decrease in the superior longitudinal fasciculus, and around 16% increase in the forceps regarding the scale factor. Among the fiber bundles, there is also an increase of up to 9% in the forceps and a decrease of up to 12% in the superior longitudinal fasciculus considering the shape factor. From the deviations of the shape factors from different fiber bundles, we can expect potential AFD deviations that could be resulted from the intrinsic mismatch of the response functions. When considering the potential merging or separation of FOD peaks that could occur as a result of the shape factor deviations (Figure 5), the AFD deviations could be disproportionately larger than the shape factor changes.

### FOD characteristics in relation to the response function modeling

In this study, we modeled the response function with the basic tensor model in simulations, and fit a tensor to the recursive calibrated response function for in vivo data to evaluate the shape and scaling factor. The tensor model allows us to represent the response function by the shape and scaling factor, which can be inversely related to the FOD characteristics through spherical harmonic basis given a set of diffusion‐weighted signals. For the in vivo experiments, the tensor‐fitting as well introduces bias in the FOD estimation as shown in Figures 7 and 8. The angular deviations are small in most brain areas, while in some voxels containing crossing fibers, there can be 1˚–3˚ angular differences between the FOD peaks determined with the recursive calibrated response function and those obtained by tensor‐fitting to the recursive calibrated response function. The percentage difference of AFD is around 36% between the FODs from the recursively calibrated response function and the tensor‐fitting response function, which resulted from the scaling difference between the response functions due to the signals used in the response calibration and the actual FOD estimation. The response function was calibrated from the data at *b* = 1000 s/mm^2^, where the Gaussian assumption typically holds, then the determined shape and scaling factor were also used for the data at *b* = 3000 s/mm^2^. While this introduced discrepancy between the scaling of the recursive calibrated response and the tensor‐fitting response, it provided an option to build a reference value of the shape factor in the FOD estimation. The AFD also shows heterogeneous deviations across the brain, up to 5%, which can be the result of a slight difference in the shape factors between the response functions.

### FOD characteristics in multishell data analysis

Multishell diffusion MRI data are becoming increasingly available in SD methods, and several studies have generalized CSD and other frameworks to accommodate multishell data.^47^, ^48^ The advantage of multishell SD includes enabling to reduce spurious FOD peaks in regions like the cortex and differentiate more tissue types.

In Figure 3, multishell CSD shows less angular deviations compared to single‐shell CSD FOD estimation. The angular deviations are mainly affected as well by the SNR level, and slightly increase when the shape factor is getting bigger. However, there are several factors other than the shape factors and radial diffusivities of the response functions that could have caused the difference in the angular deviations, which cannot be excluded or disentangled. First, multishell data in the simulations include more gradient sampling, which entangle different angular resolutions and sensitivity to different components of the in‐vivo microstructure. More gradients on a single‐shell sampling could as well increase the angular accuracy and robustness of CSD to the response function changes. Second, the accuracy and precision of multishell CSD estimation itself are different from single‐shell CSD estimation, which can be affected by the parameters like the weight of inner‐shells and the modeling of non‐white matter tissue types. Previous studies explored the related issues^47^, ^48^, ^49^, ^50^ on multishell SD methods. Further, AFD is as well dependent on *b*‐values, as shown in a recent study,^51^ which complicates comparisons of AFD across *b*‐values.

Single shell data are well worth to be investigated especially for typical data sampling of *b*‐values in SD methods. Therefore, in the study, we focused on the suggested SD *b*‐values^30^ for the effects of shape and scale factors of the response functions.

### Limitations and future directions

When estimating AFD as an integration of each “lobe,” the scale and shape effects are not independent. Changing the shape parameter has an impact on the mean amplitude of the response functions. An increase in the shape factor of the response function, that is, a narrower response function, will reduce the overall size of the response, and thus cause an increase in the mean FOD. Vice versa, a decrease in the shape factor means a broader (and hence larger) response, and will result in a decrease in the mean FOD. Applying the current formulations of the shape and scaling factor does not allow clean separations of the impact of shape and scaling effects on AFD. A more advanced formulation of the shape factor will benefit further studies to cleanly separate its impacts on FODs from the scaling factor effects.

The reference value of the shape and scaling factor of the simulated diffusion‐weighted signals matches with the values in the corpus callosum as reported before. However, recent studies^45^, ^52^, ^53^, ^54^ indicated that the diffusivities of fiber bundles in the brain are not always the same. There is not a full map of diffusivity characteristics of each white matter structure yet. Although our simulation study included the same configurations of crossing fiber bundles in a voxel, in reality, the diffusivities of these crossing fibers may not be identical.

In this study, we showed tractography results of an HCP subject using the tensor‐fit to the recursively calibrated response function and modified response functions. In group studies between healthy subjects and patients with neural degradation diseases (e.g., Alzheimer's disease), applying the group response function in the FOD estimation is suggested, as otherwise the FOD characteristic changes could be absorbed into the alterations of response functions, leading to failures of discovering FOD metrics changes.

Overall, the study demonstrates with numerical simulations and in vivo HCP data that decreasing the shape factor of the response function can cause large angular deviations of the FOD peak orientations in crossing fibers. Sharper response functions are responsible for introducing spurious peaks, which can also confound subsequent tractography results. Extremely low shape factors of the response function can cause significant angular deviations and may complicate the interpretation in studies involving pathology. In addition, although individual angular deviations of FOD peak orientations are small for single voxels at most separation angles, the adverse effect can accumulate for brain tractography. Since smaller separation angles are more sensitive to changes of response function shape factors, future work of intersubject AFD and pathological groups should be aware of this possible confounding factor when investigating brain structures with crossing fiber configurations.
